# Effects of Diagnostic Errors in Pattern Differentiation and Acupuncture Prescription: A Single-Blinded, Interrater Agreement Study

**DOI:** 10.1155/2015/469675

**Published:** 2015-04-07

**Authors:** Ingrid Jardim de Azeredo Souza Oliveira, Arthur de Sá Ferreira

**Affiliations:** Laboratório de Simulação Computacional e Modelagem em Reabilitação, Programa de Pós-graduação em Ciências da Reabilitação, Centro Universitário Augusto Motta (UNISUAM), 21041-010 Rio de Janeiro, RJ, Brazil

## Abstract

This study compared the interrater agreement for pattern differentiation and acupoints prescription between two groups of human patients simulated with different diagnostic outcomes. Patients were simulated using a dataset about* zangfu *patterns and separated into groups (*n* = 30 each) according to the diagnostic outcome determined by a computational model. A questionnaire with 90 patients was delivered to 6 TCM experts (4-year minimal of clinic experience) who were asked to indicate a single pattern (among 73) and 8 acupoints (among 378). Interrater agreement was higher for pattern differentiation than for acupuncture prescription. Interrater agreement on pattern differentiation was slight for both groups with correct (Light's *κ* = 0.167, 95% CI = [0.108; 0.254]) and incorrect diagnosis (Light's *κ* = 0.190, 95% CI = [0.120; 0.286]). Interrater agreement on acupuncture prescription was slight for both groups of correct (*ι* = 0.029, 95% CI = [0.015; 0.057]) and incorrect diagnosis (*ι* = 0.040, 95% CI = [0.023; 0.058], *P* = 0.075). Diagnostic performance of raters yielded the following: accuracy = 60.9%, sensitivity = 21.7%, and specificity = 100%. An overall improvement in the interrater agreement and diagnostic accuracy was observed when the data were analyzed using the internal systems instead of the pattern's labels.

## 1. Introduction

Diagnostic errors are difficult to recognize but are not rare in the Western practice [1]. The difficulty for differentiating between two closely related or similar diagnoses, with possibly very different prognosis or therapeutic options, is acknowledged as a source of error [2, 3]. Traditional medicines, in particular traditional Chinese medicine (TCM), are no exceptions. The systematic-philosophic relationships between humans and nature applied by TCM experts [4] do not guarantee an error-free diagnostic process [5]; different patterns that require distinct therapeutic choices regarding acupuncture prescription might also be confused. In contrast with the available treatment regimens for various diseases in the Western medicine, there are no defined protocols of acupoints for patterns mainly because of both the personalized approach of TCM's diagnostic process and the large variety of criteria for selecting acupoints [6]. In this sense, high interrater agreements, that is, the degree to which raters achieve identical results under similar assessment conditions rating the same items [7], alongside an accurate diagnosis are two important characteristics of diagnostics models.

Previous studies reported a variable degree of interrater agreement on pattern differentiation and/or therapeutic prescription [8–16], though they present important limitations either from the TCM or scientific perspectives. For instance, there was a lack of calculating and/or reporting statistics of agreement [9–11, 13] or a lack of investigating the pattern differentiation and therapeutic prescription for the same cases [10, 12–16]. Most importantly, all the above-cited studies used real human patients with a narrow range of diseases and corresponding TCM patterns, in which the true patterns were unknown and, therefore, it was not possible to assess the diagnostic accuracy of TCM experts with a gold-standard model. However, no study investigated the magnitude of the influence of diagnostic errors on the agreement of TCM experts for pattern differentiation and therapeutic prescription.

Our group developed automated systems to study the pattern differentiation process. The manifestation profile simulation algorithm (MPSA) was proposed [17] for simulation of cases and controls with known diagnosis; the current version of MPSA is implemented in the* SimTCM* model [18, 19]. The pattern differentiation algorithm (PDA) was introduced in the same study [17] and its current version [20] applies two objective criteria, explained (*F*_%_) and available information (*N*_%_), respectively, for selection of candidate patterns and ranking them as diagnostic hypothesis. Both PDA and MPSA were used to investigate diagnostic errors in pattern differentiation; because the true target-pattern of human patients was known from the* SimTCM* and were paired to the diagnosis obtained from the PDA [5], the diagnostic outcomes were separated into correct diagnosis, misdiagnosis, or no diagnosis. Using the four examinations, the lowest misdiagnosis and no diagnosis rates among 73* zangfu* patterns were observed and shared manifestation among dual patterns was identified as an important source of error in pattern differentiation [5].

Assessing the interrater agreement of TCM experts for pattern differentiation and acupuncture prescription under different diagnostic outcomes might provide new insights about the causes and/or consequences of diagnostic errors in this traditional medical practice. Therefore, this study investigated the effects of diagnostic outcomes on the interrater agreement for pattern differentiation and acupoint prescription. More specifically, this study compared the interrater agreement of TCM experts for pattern differentiation and acupoints prescription between two groups of simulated patients with different diagnostic outcomes.

## 2. Material and Methods

### 2.1. Study Design

**Figure 1:**
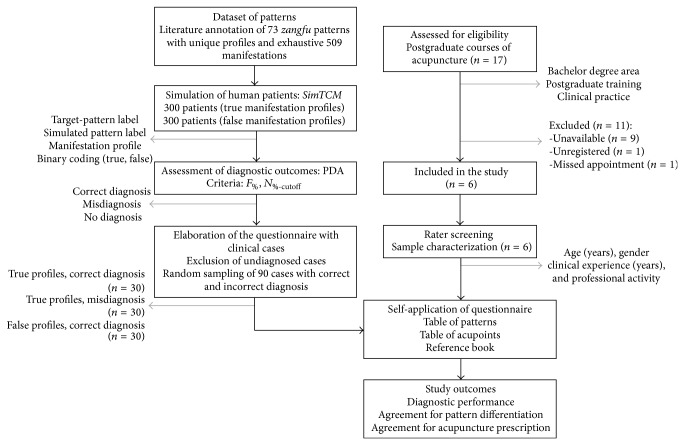
Study flowchart.

This was an observational, cross-sectional study that followed the Guidelines for Reporting Reliability and Agreement Studies (GRRAS) [7]. This study also followed the STandards for Reporting Interventions in Clinical Trials of Acupuncture (STRICTA) [21, 22] collect data regarding the characteristics of the sample of TCM experts enrolled in the study.  Figure 1 exhibits the study flowchart. Raters were blinded regarding the results of the other raters enrolled in this study.

### 2.2. Ethics

The institutional ethics committee approved this study prior to its execution (CAAE: 35723214.6.0000.5235). All participants signed an informed consent form to participate in this study after being informed about the study aims, potential risks, and benefits related to their participation.

### 2.3. Raters Screening and Admission

Raters were independently assessed for eligibility from postgraduate courses in acupuncture at local institutions nearby the Rio de Janeiro city (RJ, Brazil). Raters who simultaneously met the following criteria were included in the study: (1) bachelor degree in any health-related course recognized by the National Ministry of Education; (2) postgraduate training in acupuncture registered at the respective professional council; (3) clinical practice for at least one year; (4) signature of the informed consent form after reading about the objectives, potential risks, and benefits for participating in this research. All raters enrolled in this study answered* in loco* a self-administered questionnaire about their personal and professional characteristics for sample characterization.

### 2.4. Dataset of Patterns

The database of patterns consisted of 73* zangfu* patterns developed for a previous work, presenting 509 unique manifestations separated from examination method: inspection (*n* = 103, 20.2%), auscultation-olfaction (*n* = 31, 6.1%), inquiry (*n* = 349, 68.6%), and palpation (*n* = 26, 5.1%). The consistency and quality of the database was computationally tested before simulation of human patients to ensure that patterns were mutually exhaustive and had no duplicated manifestations describing the same pattern [5].

### 2.5. Simulation of Human Patients

Patients were simulated by selecting a variable and random number of manifestations considering all four examinations using the* SimTCM* model [18, 19]. For this simulation, it was assumed that the probability of each manifestation and pattern in the general population follows a uniform probability mass function. No user intervention was required other than the initial setup. The set of manifestations in a given pattern have mutual relationships due to the same or similar pathogenesis. Likewise, different patterns might share manifestations due to the same or similar etiology. This mutual relationship between the randomly selected manifestations for a given pattern was ensured by selecting the manifestations for a given pattern, either *k* or* q*, from the same* pattern* as described in the dataset. It is worth noticing that the dataset was developed to simultaneously maintain the internal relationship among manifestations of a given pattern and the cooccurrence of the same manifestation among different patterns.

The* SimTCM* model output a TXT file used in the subsequent stages of this research with the following data: (1) the label of the target-pattern under simulation; (2) the label of the pattern randomly selected for simulation; (3) the manifestation profile as comma-separated values; and (4) the binary code representing the patient as “1” or “0” if the simulated patient corresponds to either a true profile (target-pattern = simulated pattern) or a false profile (target-pattern ≠ simulated pattern), respectively. A total of 300 pairs of true and false manifestations profiles were simulated among all 73 patterns.

### 2.6. Assessment of Diagnostic Outcomes of the Simulated Sample of Patients

To objectively test whether the simulated patient could have its pattern differentiated (either correctly or not) among several candidates in a dataset, an automated model for pattern differentiation must be used. PDA was chosen among other automated methods because it is the most accurate automated method for pattern differentiation considering a large set of patterns regardless of an underlying medical disease [23].

Pattern differentiation of the sample of simulated patients was performed by the PDA using its both criteria *F*_%_ and *N*_%-28.5%_ for separation into diagnostic outcomes. PDA's diagnostic performance is high for simulated patients (accuracy = 94.7%; sensitivity = 89.8%; specificity = 99.5%) [20]. PDA is used to input the manifestation profiles from the above-cited TXT file and output the identified pattern. The diagnostic outcomes were obtained by comparing the label of the simulated pattern by* SimTCM* with the label of the identified pattern by PDA, yielding one of these three outcomes: “correct diagnosis” (simulated pattern = identified pattern), “incorrect diagnosis” (simulated pattern ≠ identified pattern), or “no diagnosis” (no pattern was identified as a diagnosis by PDA) [5]. Simulated patients with true and false profiles were separated into categories of correct and incorrect diagnostic outcome. Simulated patients with the no diagnosis outcome were excluded from subsequent stages because they lack a pattern label identified from PDA for comparison with TCM experts. Likewise, false profiles with incorrect diagnosis were excluded from the subsequent stages because they do not characterize a true-negative condition (i.e., false profiles with a correct diagnosis). Therefore, false profiles with incorrect diagnosis do not contribute to the analysis of diagnostic accuracy using the 2 × 2 contingency table.

### 2.7. Elaboration of the Questionnaire with Clinical Cases of Simulated Human Patients

Sixty simulated patients were randomly selected among the true profiles for each of the two diagnostic outcomes, being 30 with correct diagnosis and other 30 with incorrect diagnosis. Other 30 patients were randomly selected among the false profiles with correct diagnosis, summing up 90 patients to compose the questionnaire. More explicitly, a true profile with incorrect diagnosis represents a patient with a given target-pattern who was (mis)diagnosed with another pattern. Such a kind of manifestation profile is required to test the ability of the rater to correctly identify the target-pattern under conditions in which the automated method PDA failed. On the contrary, a false profile with correct diagnosis comprises a patient without the target-pattern who was (mis)diagnosed with the target-pattern. This kind of data is necessary to test the ability of the rater to correctly exclude the target-pattern under conditions in which the automated method PDA failed again.

All questions were prepared and presented clearly, avoiding dubious interpretation. The 90 patients were then randomly distributed into 30 groups of 3 cases each to avoid concentration of outcomes and thus providing a better distribution of diagnostic outcomes within the questionnaire.

### 2.8. Face-to-Face Interview of TCM Experts Using the Questionnaire

The hardcopy of the questionnaire was delivered face-to-face by one author who is a certified professional (IJASO) with a two-year training period and nine years of TCM clinical experience.

The questionnaire was self-administered and the investigator did not interfere in its filling. The following material was available to the rater for usage and consulting: a hardcopy table with 74 response options (73* zangfu* patterns [24] + 1 option, “it is not possible to identify the pattern”), a hardcopy table with 379 response options (361 channel acupoints [25] + 17 miscellaneous acupoints [6] + 1 option, “no acupoint”), and the reference book used for the construction of database standards with their manifestations [24]. The raters were allowed 10 minutes to familiarize themselves with the tables containing the response options, though no time limit will be posed to complete the form. To reproduce clinical conditions, the rater was allowed to consult the reference book but was not encouraged to. Only one answer was required for questions about the diagnosis, whereas between 1 and 8 answers for questions were required about the acupoints. An independent response form was provided for each rater for filling the respective code regarding the identified pattern and the prescribed acupoints for each simulated patient in the questionnaire.

### 2.9. Raw Data Tabulation and Synthesis

Data analysis was conducted at the Laboratory of Computational Modeling and Simulation in Rehabilitation (RJ, Brazil) after data collection from all raters. The rater's responses to each patient were paired to the numbering of the questions. The coding for responses about the specific 74 options for diagnosis and 379 options for prescription was typed into an electronic worksheet by one researcher (IJASO) and checked by the other one (ASF), also paired to the numbering of the questions.

Because a specific* zangfu* pattern's label is composed by several pieces of information regarding the morbid condition itself (i.e., the nature, the location, and the affected internal organ or channel), a more general set of codes was provided regarding the affected internal system. By doing this, it was possible to investigate this study's outcome regarding specific and general aspects of TCM pattern differentiation. Therefore,* zangfu* patterns were coded according to the affected* zangfu* itself: heart (*xin* = 1), liver (*gan* = 2), spleen (*pi* = 3), lung (*fei* = 4), kidneys (*shen* = 5), pericardium (*xinbao* = 6), small intestine (*xiaochang* = 7), gallbladder (*dan* = 8), stomach (*wei* = 9), large intestine (*dachang* = 10), bladder (*pangguang* = 11), and triple energizer (*sanjiao* = 12). Because more than one* zangfu* system was affected in the same pattern (e.g.,* fei-pi-shen* to generate* tanyin*), they were combined as separate codes: liver-gallbladder (*gan-dan* = 13), stomach-spleen (*wei-pi* = 14), kidney-lung (*shen-fei* = 15), kidney-heart (*shen-xin* = 16), lung-spleen-kidney (*fei-pi-shen* = 17), and lung-spleen-kidney-xin (*fei-pi-shen-xin* = 18). Acupoints were coded with the same sequence as the internal organs for correspondence with the specific channels (codes 1 to 12 only); additional codes were provided for the governing vessel (*dumai* = 19), conception vessel (*rename* = 20), extra-channel acupoints (=21), and no acupoints (=22).

Parameters related to the diagnostic performance of each rater were obtained from 2 × 2 contingency tables [23] made from the comparison between the results of the simulation by* SimTCM* (gold-standard method) and the pattern differentiation by each rater:

- true positives (*TP*): manifestation profiles simulated with the target-pattern that were correctly identified by the rater as present;
- false negatives (*FN*): manifestation profiles simulated with the target-pattern that were erroneously identified by the rater as absent;
- false positives (*FP*): manifestation profiles not simulated with the target-pattern that were erroneously identified by the rater as present;
- true negatives (*TN*): manifestation profiles not simulated with the target-pattern that were correctly identified by the rater as absent.

### 2.10. Statistical Analysis

Data analysis was reported based on single measurements of each rater. Values were presented as median [minimum; maximum] for continuous variables, and absolute and relative frequencies (%) for categorical variables. Histograms of the empirical values obtained for the coefficients of agreement under the bootstrap procedure were generated. All statistics were grouped by diagnostic outcome, considering the true profiles with correct and incorrect pattern differentiation as performed by PDA. The value of statistical significance is *P* < 0.05 (one-tailed tests).

The Light's *κ* (kappa) coefficient [26] recommended for studies with fully crossed design in which all cases are classified by multiple raters [27] was calculated, grouped by diagnostic outcome. Janson and Olsson's *ι* (iota) coefficient [28] recommended for studies with multivariate analysis by multiple raters on the same participants was calculated for testing the agreement regarding acupuncture prescription. The 95% confidence interval (95% CI) was calculated using the bootstrap procedure and bias-corrected accelerated method (BCa) with* B* = 1,000 replications [29]. Empirical *P* values were calculated using (*r* + 1)/(*B* + 1), where *r* is the number of replications that produce greater than or equal to that calculated with statistical data [30]. Null hypotheses for group with correct diagnosis comprised *κ* = 0 and *ι* = 0 (agreement not better than chance), whereas the null hypotheses for the group with incorrect diagnosis comprised *κ*_incorrect_ = *κ*_correct_ and *ι*_incorrect_ = *ι*_correct_ (no difference between correct and incorrect diagnoses) for pattern differentiation and acupuncture prescription, respectively. Both coefficients of agreement were qualitatively interpreted as poor (<0.00), slight (0.00 to 0.20), fair (0.21 to 0.40), moderate (0.41 to 0.60), substantial (0.61 to 0.80), or almost perfect (0.81 to 1.00) [31].

Accuracy, sensitivity, specificity, positive, and negative predictive values were calculated for both single rating and grouped data [32, 33]. Accuracy was tested using a binomial procedure to assess if this rate was better than chance (i.e., no information rate, *H*_0_ = 50%).

The Spearman's *ρ* correlation coefficient [34] was used to analyze the association between control and outcome variables. The correlation values and their qualitative levels were described as: no association (0.00), negligible association (±0.01 to ±0.20), weak association (±0.21 to ±0.40), moderate association (±0.41 to ±0.70), strong association (±0.71 to ±0.99), and perfect association (±1.00) [35].

The major factor that influences the power of statistical analysis is the number of cases; it is not advantageous to increase the number of raters because the effects on the statistical power and amplitude of confidence intervals are small [36]. It was demonstrated that as the number of raters increases the required number of cases diminishes, although the saving in sample size rapidly decreases after five raters [37]. Therefore, a sample size of five raters was required to investigate 30 patients per category. Because the available formulas for determining the sample size consider up to 5 outcome categories (and our data contained 73 categories for diagnosis), the following procedure was adopted. A separated routine was written to derive samples sizes for 2, 3, 4, and 5 outcome categories considering the confidence interval perspective and parameters set to *κ*_0_ = 0.75, 95% CI = [0.61; 0.80], and five raters. The obtained sample sizes for these outcome categories were then fitted to an exponential model, adjusted as *n* = 400.1789 · category^−0.622923^,  (*R* ^2^ = 0.942), where *n* is the required sample size. Using the fitted model for extrapolation to the number of patterns (=73), the minimum sample of 28 simulated patients per group was required.

### 2.11. Computational Resources

A computer with 2.26 GHz Intel Core 2 Duo microprocessor with 2 GB RAM running Mac OS X 10.10 (Apple Inc., USA) was used for data tabulation and analysis. The* SimTCM* and PDA algorithms were implemented as independent computational routines in LabVIEW 2014 (National Instruments, USA) running on Windows Vista compilation (Microsoft Corp., USA). Data from raters were tabulated in an electronic worksheet in Excel for Mac 2011 (Microsoft Corp., USA) using automatic data validation. Statistical analysis was performed in* R* 3.1.1 [38] using “boot” [39], “caret” [40], “irr” [41], “kappaSize” [42], “psy” [43], and “xlsx” [44] packages using customized routines for data reading and analyses. Randomizations were performed using an online pseudorandom generator of numbers and sequences (http://www.random.org). Computational routines were developed and implemented by the same researcher (ASF) who is a certified professional with a two-year training period and 13 years of TCM clinical experience. All tables with raw data and computational routines for data analysis in *R* language are freely available from the authors upon request by e-mail.

## 3. Results

Seventeen TCM raters were independently assessed for eligibility between November 2014 and December 2014. Eleven raters were excluded because they were unavailable during the period for data collection (*n* = 9), not yet registered in the professional council (*n* = 1), or missed the appointment after two scheduling (*n* = 1). Six raters (all physiotherapists, one also which is a medical doctor) were included in the study and filled in both questionnaires (Table 1). All raters stated that they perform pattern differentiation before acupuncture treatment selection in their daily professional activity.

**Figure 2:**
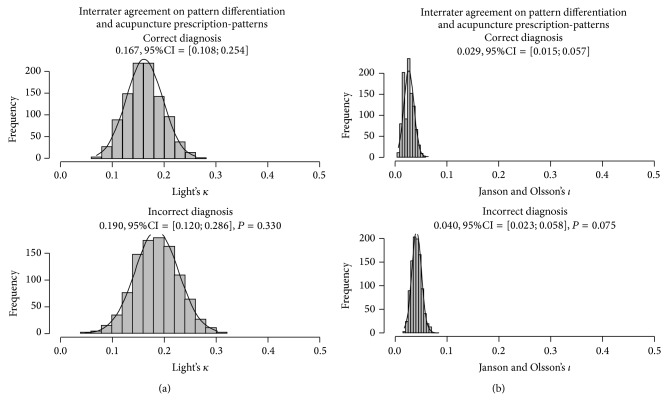
Bootstrap analysis of interrater agreement on pattern differentiation (a) and acupuncture prescription (b) estimated from the specific labels of 73* zangfu* patterns of simulated human patients grouped by correct (upper row, *n* = 30) or incorrect (lower row, *n* = 30) diagnosis.

Figure 2 exhibits the empirical histograms for the bootstrap resampling of *κ* and *ι* considering the* zangfu* patterns coded by their specific labels. In general, interrater agreement was higher for pattern differentiation than for acupuncture prescription. A significant, slight interrater agreement on pattern differentiation was observed for simulated patients with the correct diagnosis outcome (*κ* = 0.167, 95% CI = [0.108; 0.254], *P* < 0.001). No significant difference in interrater agreement was observed for simulated patients with incorrect diagnosis outcome (*κ* = 0.190, 95% CI = [0.120; 0.286], *P* = 0.330). Likewise, a significant slight significant interrater agreement for acupuncture prescription was observed for the group of simulated patients with correct (*ι* = 0.029, 95% CI = [0.015; 0.057], *P* < 0.001) diagnostic outcome, although no significant statistical change was noticed for the group with the incorrect diagnosis outcome (*ι* = 0.040, 95% CI = [0.023; 0.058], *P* = 0.075).

**Figure 3:**
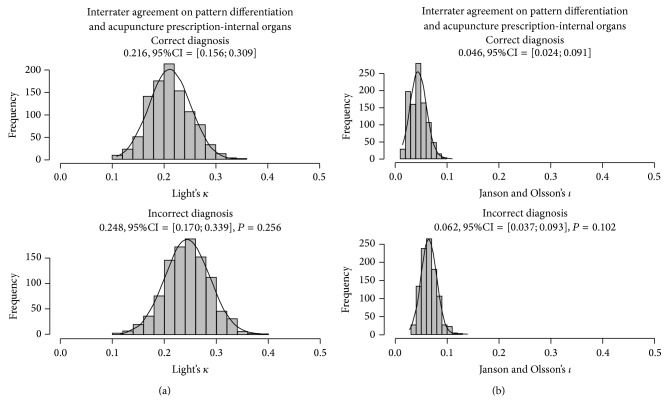
Bootstrap analysis of interrater agreement on pattern differentiation (a) and acupuncture prescription (b) estimated from the affected* zangfu* systems of simulated human patients grouped by correct (upper row, *n* = 30) or incorrect (lower row, *n* = 30) diagnosis.

An overall improvement in the interrater agreement was observed when the simulated patients were analyzed using the codes for the internal systems (Figure 3); again, interrater agreement was higher for pattern differentiation than for acupuncture prescription. A significant, fair interrater agreement on pattern differentiation was observed for simulated patients with the correct diagnosis outcome (*κ* = 0.216, 95% CI = [0.156; 0.309], *P* < 0.001). No significant change in interrater agreement was observed for simulated patients with incorrect diagnosis outcome (*κ* = 0.248, 95% CI = [0.170; 0.339], *P* = 0.256). Nonetheless, a significant yet slight interrater agreement for acupuncture prescription was observed for either group of simulated patients with correct (*ι* = 0.046, 95% CI = [0.024; 0.091]) and incorrect (*ι* = 0.062, 95% CI = [0.037; 0.093], *P* = 0.102) diagnostic outcome, again without statistical significance between diagnostic outcomes.

Table 2 summarizes the results of the diagnostic performance of each rater, as well as the group summary. The diagnostic performance of the raters for identifying* zangfu* patterns with correct diagnosis showed that only 2 raters (33%) performed pattern differentiation better than chance (accuracy = 60.9% [56.7; 70.0]). Sensitivity was low (21.7%), whereas specificity was high (100%); positive predictive values were also high (100%), with low negative predictive values (56.1%). Similar results were observed for identifying* zangfu* patterns with incorrect diagnosis according to PDA. Two raters (33%) performed pattern differentiation better than chance (accuracy = 58.3% [51.7, 63.3]) and presented low sensitivity (16.7%), high specificity, and positive predictive values (100%) and low negative predictive value (54.5%).

An overall improvement in the diagnostic performance was also observed for the pattern differentiation using the coding by the affected internal system. Five raters (83%) performed pattern differentiation better than chance in cases with correct diagnosis (accuracy = 66.7% [55.0; 73.3]). Again, sensitivity was low (36.7%) and specificity conversely high (93.4%). Positive predictive values were also high (88.0%), with low negative predictive values (60.5%). Regarding patients with incorrect diagnosis, the same five raters (83%) performed pattern differentiation better than chance (accuracy = 67.5% [58.3, 75.0]) and presented low sensitivity (38.3%), high specificity, and positive predictive values (93.4% and 88.3%, resp.) and low negative predictive value (61.1%).

The association analysis regarding the correct diagnostic outcome showed no significant positive correlations of diagnostic accuracy with being a postgraduate course professor (*ρ* = 0.399, *P* = 0.217), time since postgraduate (*ρ* = 0.277, *P* = 0.297), or age (*ρ* = 0.087, *P* = 0.435). No significant negative correlations were also observed between diagnostic accuracy and being supervisor of a clinic-school (*ρ* = −0.396, *P* = 0.218) or sex (*ρ* = −0.105, *P* = 0.422). No significant positive correlations were also observed for diagnostic accuracy and being a postgraduate course professor (*ρ* = 0.399, *P* = 0.217), time since postgraduate (*ρ* = 0.277, *P* = 0.297), or age (*ρ* = 0.232, *P* = 0.329). No significant negative correlations were observed between diagnostic accuracy and sex (*ρ* = −0.315, *P* = 0.272) or the role of supervisor of a clinic-school (*ρ* = −0.198, *P* = 0.353).

## 4. Discussion

This study investigated the effects of diagnostic outcomes on the TCM interrater agreement for pattern differentiation and acupoint prescription using both specific and general characteristics of patterns. The main findings of our study comprised the following: (1) interrater agreements on pattern differentiation and acupoint prescription were slight regardless of whether the diagnosis was accurate or not, (2) interrater agreement was fair for differentiating combinations of affected internal organs, although it remained slight for acupuncture prescription, (3) diagnostic accuracy of TCM raters for differentiating* zangfu* patterns was not better than chance for most raters, although it was better for differentiating the affected internal organs, and (4) no significant effects of personal and professional variables were detected. To the best of our knowledge, this is the first study to simultaneously assess the interrater agreement for pattern differentiation and acupuncture prescription in specific and general TCM theories, along with the possible effects of the diagnostic errors in these outcomes.

The observed low agreement for TCM diagnosis may be due to several factors, particularly related to TCM theories and methodological aspects of this study, which are discussed in a separated section. As related to TCM theories, the systematic-philosophic relationship used for pattern differentiation tries to provide clear distinctions among all* zangfu* patterns [4, 6]. However, it does not guarantee the occurrence of pathognomonic manifestations; several manifestations are shared among patterns and, therefore, may confuse the TCM expert when diagnosing a patient [5]. The lack of international standards for describing each pattern's manifestation and no training before filling in questionnaires are another two potential sources of variability in diagnosis, albeit the latter condition is a close representation of the clinical scenario. Nonetheless, our results are consonant with previous ones investigating TCM agreement on diagnosis of specific* zangfu* patterns: slight interrater agreement (unspecified *κ* = 0.11; four raters) [8]; interrater agreement below chance expectations (*κ* values not available; three raters) [9]; “little” agreement (*κ* values not available; three raters) [11]; slight interrater agreement (Fleiss' *κ* = 0.112; four pairs of raters) [12]; and slight interrater agreement (*κ* in range 0.014 to 0.179, eight raters) [15]. In contrast, our results were lower than those observed by Xu et al. [16], who reported slight to almost perfect interrater agreement (Cohen's *κ* in range 0.005 to 0.801; two raters). However, the raters applied very specific diagnostic criteria that might have helped them achieve so high agreements. Our observations that interrater agreement was better for more generic aspects of TCM diagnosis and prescription are also similar to others studies: interrater agreements varying between slight (*κ* = 0.15) and almost perfect (*κ* = 0.87) among three raters [13] and moderate interrater agreement (Cohen's *κ* = 0.56; two raters) [14].

It is worth noticing that the above-cited studies focused on the paradigm of “pattern|disease” [45], in which few TCM patterns are studied within the context of a given disease at any stage of its natural history: irritable bowel syndrome [8], rheumatoid arthritis [9], frequent headaches [11], mixed sample of healthy participants and patients with chronic diseases [12], hypercholesterolemia [13], prediabetes [14], fertile and infertile women [15], and cardiovascular diseases [16]. This approach limits their external validity to a more general interpretation of the TCM practice. Therefore, since our study did not specify an underlying disease and patterns were randomly selected to compose the simulated sample of patients our findings are considered as representative regarding both specific* zangfu* patterns and affected internal organs.

The observed low agreement for acupuncture prescription may be explained mainly by the likewise low agreement in pattern differentiation itself, along again with other unique aspects of acupuncture practice. There are plenty of criteria for selecting and combining acupoints: local, distant, specific, painful, and* yin-yang* combinations; balanced and imbalanced combinations are among the most commonly used [6, 24]. In addition, each acupoint has several therapeutic indications that might be partially shared between either adjacent or distant acupoints, on the same channel or not [6, 24]. Our results are also consonant with two above-cited studies that also investigated TCM agreement on prescription and reported slight interrater agreement on herbal therapy (*κ* = 0.16; four raters) [8] and “little” agreement on acupuncture prescription (*κ* values not available; three raters) [11]. Using data provided from Coeytaux et al. [11], in which 37 patients with frequent cephalea were interviewed by three TCM raters, and our methods described in this study, we found a slight agreement on acupuncture prescription considering either the acupoint's label (*ι* = 0.028 [0.016; 0.042]) or the acupoint's channel (*ι* = 0.026 [−0.001; 0.047]).

It is worth discussing our findings in light of two famous principles related to the personalized approach of TCM for diagnosis and treatment. On the one hand, the traditional statement “different treatments for the same pattern” [4] acknowledges that it is not an error to treat the same pattern with a variety of acupoint sets. This statement holds because the implicit information herein is that the same pattern also manifests as different collections of signs and/or symptoms in each person due to personal and environmental factors [6]. Hence, it is possible that different treatments are selected for the same pattern depending on the patient's manifestation profile and it should not be considered as a source of error. On the other hand, another traditional statement, “the same treatment for different patterns” [4], acknowledges that to apply the same acupoints' set to treat different patterns is not an error. This statement also holds because of another implicit information: each acupoint presents different collections of therapeutic actions [6]. It is indeed possible that the same acupoints' set is indicated for different patterns depending on their expected therapeutic action and thus it should not be considered as a source of error for prescription. Because our sample of TCM raters was not aware about the true diagnosis of each patient and the automated “simulation-identification” procedure guaranteed that each manifestation profile corresponded to a unique diagnosis, the observed slight agreements for pattern differentiation and acupoint prescription are not related to these statements.

The lower diagnostic accuracy of TCM raters regarding the simulated sample with the correct diagnosis identified by PDA was expected under both specific and general aspects of diagnosis; while raters relied on subjective data analysis, the PDA applied objective criteria for constructing a list of diagnostic hypothesis and selecting a diagnosis. Such a low accuracy of the TCM raters was accompanied by a low sensitivity and a high specificity, which indicates that raters are less capable of correctly identifying the true pattern as present but are more capable of correctly identifying the true pattern as absent. Therefore, the subjective criteria adopted by TCM raters to perform pattern differentiation seem to act mainly to exclude unlikely diagnostic hypotheses rather than to provide the true one, which in turn also helps us explain the low interrater agreement for pattern differentiation. Because literature showed that the interrater agreement on pattern differentiation might be significantly improved after supervised practice [8, 10, 12], efforts should be made directly to test whether training might also improve the diagnostic accuracy of TCM raters.

An unanticipated, interesting finding was the diagnostic accuracy under the incorrect diagnosis (i.e., false profiles). It must be emphasized that this group comprised simulated patients with a unique diagnosis (known from the* SimTCM* output) that were misdiagnosed by PDA using its quantitative criteria. PDA's misdiagnosis rate was low, nearly 6.0%, for these 73* zangfu* patterns [5]; nonetheless, this result may be interpreted as the superiority of TCM raters to perform pattern differentiation in cases that PDA failed to report a correct diagnosis. This result is encouraging but raises a new challenge: to improve the detection of misdiagnosed patterns using PDA or other automated methods for pattern differentiation.

We found no personal (age, sex) or professional (clinic or scholar activity, time since postgraduate) variable to be associated with the diagnostic accuracy. Although our study was not designed to identify association between variables, these results may be used to plan sample sizes for larger studies aiming to determine if these factors are indeed determinants of an accurate pattern differentiation, if any.

Collectively, our findings raise a novel explanation for the low interrater agreement for pattern differentiation and even lower agreement for acupuncture prescription: the nonlinear, multivariate nature of patterns and acupoints.* Zangfu* patterns can be defined by at least four vectorial dimensions (e.g.,* fei-qi* deficiency: vital substances,* qi*; internal organs,* fei*; pathophysiologic mechanisms, deficiency; and clinical manifestations) [6, 24], whilst acupoints can be defined by at least three dimensions and two scalar plus a vectorial one (e.g., LI-4* hegu*: channel number, 4; internal organ, large intestine-*dachang*; and therapeutic indications) [6, 24]. If the sharing of clinical manifestations and therapeutic indications among vectorial variables of patterns and acupoints is also considered [5], the whole model of “patient→TCM diagnosis→TCM prescription→treatment” is also a nonlinear one with two sequential stages. The input of clinical manifestations into the first stage of such a multivariate nonlinear model partially explains the variability in the outputted diagnosis because a variety of subsets of clinical manifestations may be present in the same pattern in different subjects. In sequence, inputting the diagnosis into the second stage of this multivariate nonlinear model for acupoint selection adds more variability to the outputted acupoints. Adding some “noisy information” in either or both stages, for instance, originated from the variability in rater's own knowledge, clinical experience and training, and ability to recognize manifestations or to differentiate between two similar conditions, turns into a scenario in which different raters might provide a variety of diagnostics and acupoints for the same subject. We thus encourage other researchers to investigate whether this model is of clinical value for improving TCM interrater agreement and possibly diagnostic accuracy as well.

There are limitations that need to be discussed for a proper interpretation of our results. Firstly, the presentation of cases in hardcopy questionnaires instead of real persons may be acknowledged as a potential source of variability in diagnosis, the simulation of cases being another interaction factor. However, the presentation of clinical cases is the core of TCM transmission since ancient times and, therefore, raters were familiarized with this type of presentation of clinical cases. In addition, the simulation of cases used manifestations as described in current literature and the usage of simulation procedures has been increasingly the method of choice for diagnostic analysis in TCM [18, 19]. Although the simulation procedure did not cover all possible combinations of manifestations for a* zangfu* pattern, the random sampling method provided a variety of combinations that may occur in daily clinical practice. Most importantly, the usage of advanced statistical methods for data simulation (*SimTCM*) and analysis (*κ*, *ι*, bootstrapping, and confusion matrices) is a major strength of our study in comparison to the previous ones and are strongly recommended for future studies on this subject.

## 5. Conclusions

Interrater agreement is slight for differentiating* zangfu* patterns and prescribing acupoints, regardless of whether the diagnosis is accurate or not. Interrater agreement is better for pattern differentiation but yet slight for acupuncture prescription based on the selected internal organs. Diagnostic accuracy of TCM raters for differentiating* zangfu* patterns is not better than chance for most raters, although it is better for differentiating the affected internal organs, in particular when an automated method failed to provide the correct diagnosis.

## Figures and Tables

**Table 1:** Descriptive data of the studied sample.

Variable	Value^*^
Sample size, *n*	6
Male	4 (66.7%)
Female	2 (33.3%)
Professional activity	
Clinical consultant	6 (100%)
Postgraduate professor	5 (83.3%)
Supervisor of clinic-school	3 (50.0%)
Age, years	43 [37; 64]
Formal training and practicing	
Duration of postgraduate course, years	2 [2; 2.5]
Acupuncture and TCM theory, hours	800
Acupuncture training, hours	400
Time since postgraduate, years	12 [4; 33]

^∗^Median [minimum; maximum] for continuous variables; frequency (%) for categorical variables.

**Table 2:** Diagnostic performance of six raters for pattern differentiation grouped by diagnostic outcome.

Outcome	Correct diagnosis (true profiles)						Incorrect diagnosis (false profiles)					
Descriptor	ACC [95% CI]	*P*-value	SEN	SPE	+PV	−PV	ACC [95% CI]	*P* value	SEN	SPE	+PV	−PV
Patterns												
Rater 1	61.7 [48.2; 73.9]	**0.046**	23.3	100	100	56.6	58.3 [44.9; 70.9]	0.123	16.7	100	100	54.5
Rater 2	58.3 [44.9; 70.9]	0.123	16.7	100	100	55.6	56.7 [43.2; 69.4]	0.183	13.3	100	100	53.6
Rater 3	60.0 [46.5; 72.4]	0.078	20.0	100	100	55.6	58.3 [44.9; 70.9]	0.123	16.7	100	100	54.5
Rater 4	56.7 [43.2; 69.4]	0.183	13.3	100	100	53.6	51.7 [38.4; 64.8]	0.449	3.3	100	100	50.8
Rater 5	70.0 [56.8; 81.2]	**0.001**	40.0	100	100	62.5	63.3 [49.9; 75.4]	**0.026**	26.7	100	100	57.7
Rater 6	61.7 [48.2; 73.9]	0.046	23.3	100	100	56.6	61.7 [48.2; 73.9]	**0.046**	23.3	100	100	56.6
Group-median	**60.9 [56.7; 70.0]**	**NT**	**21.7**	**100**	**100**	**56.1**	**58.3 [51.7; 63.3]**	**NT**	**16.7**	**100**	**100**	**54.5**
Internal organs												
Rater 1	65.0 [51.6; 76.9]	**0.014**	33.3	96.7	90.9	59.2	65.0 [51.6; 76.9]	**0.014**	33.3	96.7	90.9	59.2
Rater 2	61.7 [48.2; 73.9]	**0.046**	23.3	100	100	56.6	63.3 [49.9; 75.4]	**0.026**	26.7	100	100	57.7
Rater 3	73.3 [60.3; 83.9]	**<0.001**	56.7	90.0	85.0	67.5	70.0 [56.8; 81.2]	**0.001**	50.0	90.0	83.3	64.3
Rater 4	55.0 [41.6; 67.9]	0.259	23.3	86.7	63.6	53.1	58.3 [44.9; 70.9]	0.123	30.0	86.7	69.2	55.3
Rater 5	68.3 [55.0; 79.7]	**0.003**	46.7	90.0	82.4	62.8	75.0 [61.2; 85.3]	**<0.001**	60.0	90.0	85.7	69.2
Rater 6	68.3 [55.0; 79.7]	**0.003**	40.0	96.7	92.3	61.7	70.0 [56.8; 81.2]	**0.001**	43.3	96.7	92.9	63.0
Group-median	**66.7 [55.0; 73.3]**	**NT**	**36.7**	**93.4**	**88.0**	**60.5**	**67.5 [58.3; 75.0]**	**NT**	**38.3**	**93.4**	**88.3**	**61.1**

ACC: accuracy; 95% CI: 95% confidence interval; SEN: sensitivity; SPE: specificity; +PV: positive predictive value; −PV: negative predictive value; NT: not tested.
